# Complete Genome Sequence of the Proteorhodopsin-Containing Marine Flavobacterium *Dokdonia donghaensis* DSW-1^T^, Isolated from Seawater off Dokdo in the East Sea (Sea of Korea)

**DOI:** 10.1128/genomeA.00804-16

**Published:** 2016-08-04

**Authors:** Kitae Kim, Soon-Kyeong Kwon, Jung-Hoon Yoon, Jihyun F. Kim

**Affiliations:** a Department of Systems Biology and Division of Life Sciences, Yonsei University, Seodaemun-gu, Seoul, Republic of Korea; b Department of Food Science and Biotechnology, Sungkyunkwan University, Jangan-gu, Suwon, Republic of Korea; c Strategic Initiative for Microbiomes in Agriculture and Food, Yonsei University, Seodaemun-gu, Seoul, Republic of Korea

## Abstract

*Dokdonia* spp. have been used for investigating the lifestyles of proteorhodopsin-containing photoheterotrophs and for understanding marine photobiology. Here, we report the complete genome sequence of *Dokdonia donghaensis* DSW-1^T^ using the PacBio sequencing platform. It should provide a valuable resource for comparative genomic studies of marine life harboring microbial rhodopsins among others.

## GENOME ANNOUNCEMENT

Marine flavobacteria are a well-known reservoir of microbial rhodopsins. Microbial rhodopsins are light-activated pump proteins that translocate a specific ion across the bacterial membrane (1–4). Among them, proteorhodopsin translocates the proton outward, which forms an electrochemical potential. This in turn becomes the driving force for ATP generation and other energy-requiring processes (5, 6). The genus *Dokdonia* is a representative group for exploring physiological features of marine bacteria that harbor proteorhodopsins (7–11). Enhanced growth or increased survival during nutrient starvation has been observed for some proteorhodopsin-containing bacteria, including *Dokdonia* spp. (7–10). A recent study with *Dokdonia donghaensis* DSW-1, isolated from seawater between the two islands of Dokdo (12), showed an accelerated growth of the strain under light-illuminating conditions, which is suggested to be linked to enhanced vitamin B_1_ acquisition (11). This organism is aerobic, halophilic, nonmotile, free-living, and Gram-negative, and grows optimally at 30°C with 2% NaCl (12). The genome sequence of *D. donghaensis* DSW-1^T^ was previously reported by a Spanish group based on Illumia’s MiSeq sequencing platform (11). However, it remains as a draft of 19 contigs, which prompted us to determine its complete genome sequence.

DSW-1^T^ was cultivated in 50 mL of marine broth for 2 days, and genomic DNA was prepared using the GenElute bacterial genomic DNA kit (Sigma-Aldrich). A single-molecule real-time sequencing platform (PacBio RS II; DNA Link) that utilizes P6-C4 chemistry and the SMRT cell with MagBead OneCellPerWell version 1 protocol was employed to obtain the sequence reads. Filtrated raw data consisted of 88,381 reads with an average length of 14,016 bp. The SMRT analysis software including HGAP3, AHA, and Quiver was applied for *de novo* assembly, scaffolding, and gap-filling of the reads. A single contig was assembled through reiteration of the above procedure. The completed genome sequence of DSW-1^T^ comprises a 3,293,944-bp circular chromosome (376.1-fold coverage and 38.2% G+C contents) without a plasmid; 2,881 protein-coding sequences were annotated using Prodigal (13); and three rRNA operons and 45 transfer RNAs were predicted using HMMER (14) and Aragorn (15), respectively.

The previously reported discontinuous DSW-1^T^ genome sequence was predicted to be 3,223,976 bp in size and have 2,973 protein-coding genes and 40 tRNAs (11). A total of 69,968 bp was newly obtained by PacBio sequencing in this study, and they constitute 2.1% of the genome sequence length. These differences were due to highly repetitive intergenic sequences and intragenic sequences that exist in several copies. Data missing in the draft sequence include genes encoding hypothetical proteins, a 30-s ribosomal protein, a collagen triple helix repeat protein, an alpha-antigen precursor, and an outer membrane efflux protein BepC precursor. Also, the complete sequence retrieved two more copies of the rRNA operons and one more copy of *parD* that codes for an antitoxin and a gene for a plasmid stabilization protein. The precise genome sequence obtained by our study could be applied for research in understanding ecological roles of DSW-1^T^ and comparative genomic studies of myriads of marine flavobacteria.

### Nucleotide sequence accession number.

The complete genome sequence of *Dokdonia donghaensis* DSW-1^T^ has been deposited in GenBank under the accession number CP015125.
